# Mathematical modeling reveals cell differentiation processes and progenitor kinetics necessary for proper nephrogenesis

**DOI:** 10.3389/fcell.2025.1695380

**Published:** 2025-12-11

**Authors:** Matthew R. Hawkins, Stuart E. Jones, Hannah M. Wesselman, Cecilia Cesa, Joshua Moeller, Rebecca A. Wingert

**Affiliations:** Department of Biological Sciences, Center for Stem Cells and Regenerative Medicine, Center for Zebrafish Research, University of Notre Dame, Notre Dame, United States

**Keywords:** nephron, pronephros, renal progenitor, segmentation, zebrafish

## Abstract

**Introduction:**

The developing zebrafish nephron contains a diverse repertoire of cell populations with intrinsically dynamic roles and interactions. Previous studies characterizing these populations have utilized genetic approaches based on powerful molecular methods, such as *in situ* hybridization, immunohistochemistry, and both live and fixed imaging. However, our understanding of the cellular characteristics of the populations necessary for renal development in the zebrafish model is lacking in comparison to our understanding of the genetic/ morphogenic drivers of nephron segmentation.

**Methods:**

To investigate the potential drivers of nephron development from the population perspective, we incorporated traditional molecular tools for determining cellular characteristics as well as algorithmic based optimization methods. We synthesized multiple ODE based models to investigate hypotheses surrounding populations existing in early zebrafish nephron development.

**Results:**

Our model based findings showed that deferential forms of fate processes may be necessary for zebrafish pronephros development. Also, we provide evidence for differentiated populations being the potential drivers of renal development rather than progenitor populations.

**Discussion:**

Our findings further bring forward the idea that differentiated populations are vital for the growth and development at the single nephron level in the zebrafish model. We also continue the investigation of varied forms of fate being necessary in renal development.

## Introduction

The vertebrate kidney serves as the biological nexus between osmoregulation and waste secretion. Proper development of nephrons, the structural and functional units of the kidney, is crucial to perform these essential physiological tasks. The adult human kidney possesses between 200,000 and 2.7 million nephrons that govern fluid flow and facilitate waste dismissal (Charlton et al., 2021). Each nephron consists of a blood filter (the glomerulus), followed by an epithelial tubule that performs reabsorption and secretion of materials which connects to a collecting duct for drainage of urine. Renal organogenesis in vertebrates entails the progressive formation and degradation of kidneys that have increasing nephron number and architectural complexity, known as the pronephros, mesonephros, and metanephros (Romagnani et al., 2013). Higher vertebrates including humans and other mammals form all three versions, although the pronephros is vestigial and rapidly degrades during gestation. In lower vertebrates such as the zebrafish, the pronephros is functional through early larval stages, after which mesonephros emerges and ultimately becomes the final adult kidney form (Gerlach and Wingert, 2013).

**FIGURE 1:**
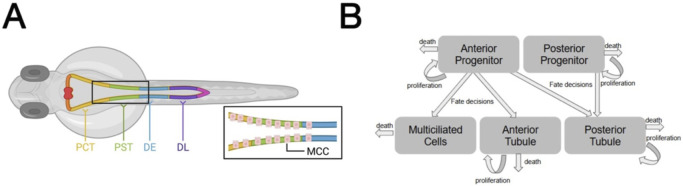
Portrayal of five distinct tubule cell populations during zebrafish nephrogenesis. **(A)** Rendering of zebrafish pronephros and associated tubules and the MCC population. The blood filter (glomerulus) is not shown. **(B)** Diagram of population dynamics in the working model. Figure created with BioRender.com.

The zebrafish pronephros has been used extensively to investigate the mechanisms of nephrogenesis because its nephron composition is well conserved with other vertebrates (Chambers et al., 2024), and there is close genetic similarity between zebrafish and humans (Howe et al., 2013). Furthermore, the zebrafish pronephros possesses a simple anatomy of two nephrons. The nephrons share a single, midline blood filter, and the tubules are situated along the trunk, enabling their easy observation during embryogenesis (Drummond et al., 1998). The pronephric tubules contain four physiologically distinct segments: the proximal convoluted tubule (PCT), the proximal straight tubule (PST), the distal early (DE) tubule, and the distal late (DL) tubule (Figure 1A) (Wingert et al., 2007). Interspersed within the three most proximal tubule populations is a population of multiciliated cells (MCCs) that regulate the flow of fluid within the tubule (Figure 1A) (Ma and Jiang, 2007; Liu et al., 2007; Wesselman et al., 2022). It is accepted that these MCCs are derived from the same precursor population as the cells forming the PCT, PST, and DE (Naylor et al., 2016). The DL is formed from a distinct progenitor population that lacks the potential to form MCCs (Naylor et al., 2016).

The transition of mesenchymal precursors derived from the intermediate mesoderm into mature MCCs and tubule populations begins during segmentation stages, at approximately 10 h post-fertilization (hpf), and continues into early larval stages (Wingert et al., 2007; Wesselman et al., 2022). Changes in these populations over time are easily traceable through reliable methods to detect cell type-specific transcripts or proteins using whole-mount *in situ* hybridization (WISH) and fluorescent *in situ* hybridization (FISH) or immunohistochemistry (IF), respectively (Wesselman et al., 2023; Marra et al., 2017). However, our understanding of progenitor populations is limited by accessibility of true progenitor markers and the subsequent inability to survey fate processes in real time.

Here, we have constructed a mathematical model to examine the relationships between progenitor and progeny during early nephrogenesis in the zebrafish model, with emphasis on determining which differential forms of fate processes are likely necessary for proper renal development, the timing of proliferation across different populations, and estimating the population counts within the progenitor pools at 10 hpf, when renal development in the zebrafish model initiates. For simplicity in our studies and to provide the opportunity for emphasis on progenitor populations, we have elected to generalize tubule populations as anterior and posterior (Figure 1B). Other populations found, such as the corpuscle of Stannius and podocytes/glomerular structures, were excluded from our models to either 1) conserve free parameters that would have to be estimated/optimized for or 2) aim for better interpretability in the tubule populations and their respective precursory populations.

This body of work contributes to our understanding of renal progenitor dynamics, where we look to add to the growing body of work performed using *in vivo* and *in vitro* models. Recent transcriptomics/spatially resolved approaches have greatly enhanced our understanding of cellular differentiation and polarity of a developing tubule (Lindström et al., 2018; Lindström et al., 2021; Achieng et al., 2025; Schnell et al., 2025). Although most of these studies demonstrate transcriptional and, increasingly, spatiotemporal resolution at the cellular granularity of mammalian nephrons, we offer theoretical insights into the development of the zebrafish nephron at a temporal resolution. In other *in silico* studies of nephrogenesis, researchers have investigated renal cell differentiation; however, they have not used or examined both division-dependent and division-independent modes of fate processes (Zubkov et al., 2015; Cook et al., 2021).

## Methods

### Animal husbandry

Zebrafish were maintained in the Center for Zebrafish Research at the University of Notre Dame. All work was conducted under protocol numbers 19-06-5412 and 22-07-7335 approved by the University of Notre Dame Institutional Animal Care and Use Committee (IACUC). All experiments were carried out using wild-type (WT) Tübingen strain zebrafish. Embryos were raised and staged as described (Kimmel et al., 1995). For all work, embryos were incubated in E3 media from fertilization, anesthetized using 0.02% tricaine, and then fixed for analysis using 4% paraformaldehyde (Westerfield and Westerfield, 2007).

### WISH, FISH, IF, and image acquisition

WISH was performed using antisense RNA probes labeled with digoxigenin (*odf3b*, *slc20a1a*, *trpm7*, *slc12a1*, and *slc12a3*) generated through *in vitro* transcription from IMAGE clone templates, as previously described (Wingert et al., 2007; Gerlach and Wingert, 2014). FISH was performed using TSA Plus Fluorescein or Cyanine kits, as described (Marra et al., 2017). Whole-mount IF experiments were completed, as previously described, on PFA-fixed embryo samples, with primary and secondary antibodies as listed (Supplementary Table S1). Proliferating cells were marked with anti-PH3 (1:200 dilution), and apoptosis was detected using anti-activated Caspase3 (1:50 dilution) (Marra et al., 2017). A Nikon Eclipse Ni microscope with a DS-Fi2 camera was used to image WISH samples. IF and FISH images were acquired using a Nikon A1R confocal microscope.

### Model development

Based on our understanding of the relationship between the progenitor and mature cell populations, we constructed an ordinary differential equation-based model, in which two progenitor populations exist: anterior (*G*) and posterior (*J*) (Table 1). The anterior progenitor population would supply the post-mitotic multiciliated cell population (*M*), anterior tubule population (*A*), and posterior tubule population (*P*), whereas the posterior progenitor population would solely supply cells to the posterior tubule (*P*) (Table 1). These cell populations would represent the state variables within the model.

**TABLE 1:** State variables within the model.

State variable	Population
*G*	Anterior progenitor
*J*	Posterior progenitor
*A*	Anterior tubule
*P*	Posterior tubule
*M*	Multiciliated cell

We developed our tubule models on a general framework in which the following differential equations applied, where we demonstrate the change within the *A* or *P* populations with respect to time (Equations 1, 2):

$$\frac{dA}{dt}=l_{A}A+f_{GA}G-d_{A}A,$$ (1)

$$\frac{dP}{dt}=l_{P}P+f_{GP}G+f_{JP}J-d_{P}P.$$ (2)

Within each of these models, the anterior and posterior tubule populations undergo proliferation (l_A_ and l_P_, respectively). As the mature tubule populations increase rapidly in size during early development and plateau at later stages, we assume that the overall proliferative rate of the population is dynamic over time, thereby necessitating equations for tubule proliferation to be flexible with respect to parameterization that governs processes within each of these populations.

The multiciliated population (*M*) is a simplified version of the anterior tubule population, with a fate process from the anterior progenitor population (f_GM_), and a fixed/non-dynamic death parameter (*d*_*M*_). However, the difference between the anterior tubule population and the MCC population is that the latter is a post-mitotic population, thereby leaving the population without a proliferation term. Therefore, the MCC population can be represented as

$$\frac{dM}{dt}=f_{GM}G-d_{M}M.$$ (3)

With respect to model composition, the progenitor populations follow the same general design as tubule populations, with proliferation, fate processes, and death represented as equations to be parameterized. For the anterior progenitor population (*G*), the equation would contain a sub-equation for proliferation (l_*G*_), fates (f_*GX*_), where X is one of the three populations that are considered to be differentiated within our model (*A*, *P*, and *M*), and death (*d*_*G*_). In some cases, where differential forms of fate processes were being analyzed, we resorted to a nomenclature of *lf*_*GX*_ to represent division-independent fate processes from the anterior progenitor population to the ‘X’ differentiated population, and we used *f*_*GX*_ to represent division-dependent/asymmetric fate processes from the anterior progenitor population to the ‘X’ differentiated population (Equation 4):

$$\frac{dG}{dt}=l_{G}G-d_{G}G-{\left(l\right)f}_{GM}G-{\left(l\right)f}_{GA}G-{\left(l\right)f}_{GP}G.$$ (4)

For the posterior progenitor population (*J*), forms similar to those of the anterior progenitor population were followed. Proliferation and death of the posterior progenitor population took an identical form to that of its anterior counterpart, with *l*_G_ governing symmetric proliferation of both populations. A generic parameter for death (*d*_*J*_) was used, which represents a non-dynamic/rate-constant death throughout our simulations. Similar nomenclature was maintained for the posterior progenitor populations with respect to fates, f_*JP*_, where *P* denotes the posterior tubule population. The same nomenclature was applied for differentiating division-independent from division-dependent/asymmetric fate processes, with (*l*) denoting division-independent fate processes of the posterior progenitor population in *lf*_*JP*_ (Equation 5):

$$\frac{dJ}{dt}=l_{G}J-d_{J}J-{\left(l\right)f}_{J}PJ.$$ (5)

For all dynamic processes (proliferation and fate processes), we refer to a given process (*K*), where *K* may represent a proliferation or fate process, as a function of time *t*. Here, *K(t)* represents the proportion of a population *X* (progenitor or undifferentiated) undergoing cellular processes (proliferation or death) at time *t*. Parameters within this Gaussian function include *K*_*X_max*_, representing the maximum proportion of cells within population *X* undergoing process *K* (proliferation or death) at a given time *t*; *K*_*X_time*_, denoting the time at which the highest proportion of cells within populations *X* is undergoing process *K*; and *K*_*X_width*_, indicating the duration over which cells within population *X* would undergo cellular process *K* (proliferation or death) (Equation 6):

$$K\left(t\right)=K_{X\_\mathrm{Max}}\times 2^{\left(-{\frac{\left(t-K_{X\_Time}\right)}{K_{X\_width}}}^{2}\right)}.$$ (6)

To determine the most appropriate functional form for modeling these dynamic processes, we evaluated several candidate forms, including exponential, logistic, piecewise linear, Gaussian functions, and even constant parameterization (e.g., single parameter for fate processes). Of these, the Gaussian functions consistently minimized the residual error and most accurately captured the observed biological dynamics. Optimization of a Gaussian process similar to that utilized allows for a unique opportunity, where the free parameters K_X_width_ and K_X_time_ can be optimized to generate (relatively) linear functions, sigmoidal functions, or relatively constant values given a sufficiently large K_X_width._ This flexibility derived from the Gaussian function allowed for parameter optimization that gave maximal biological inference into cellular processes (Hensman et al., 2013; Swain et al., 2016).

In progenitor populations, we assumed that proliferation rates are higher during early development and subsequently decrease as fate processes (likely) diminish and progenitors (likely) take on a more passive role in the development of the zebrafish nephron (Tang et al., 2017). This has also been shown in the mouse model, where cap mesenchyme cells slow their proliferation rates with advancing age (Short et al., 2014). In addition, as we aimed to focus primarily on the fate-based dynamics found within these early renal populations, we utilized generalized proliferation parameters for our modeling of progenitors. To model this process, we refer to the non-specific term for progenitor proliferation as “l_*G*_.” The proliferative maximum that the progenitor population may reach can be expressed as “l*G*_max_,” the time scale in which change may occur in progenitor proliferation is referred to as “l*G*_1_,” and the rate in which changes in progenitor proliferation may occur is referred to as “l*G*_2_” (Equation 7). We introduce a constant proliferation rate of 0.001 or 0.1% to demonstrate a basal level of proliferation in the progenitor population.

$$l_{G}\left(t\right)=.001+\frac{{\mathrm{lG}}_{\max}-.001}{{\left(1+\frac{t}{{1G}_{1}}\right)}^{lG_{2}}}.$$ (7)

### Model development for investigation of later models (Model 3)

To test alternate needs of fate processes in our follow-up to Model 3, we tested two additional model structures. The first variation of Model 3, designated as 3′, was constructed by retaining all state variable equations from Model 3, except for one change to the linking equations associated with the ODEs for *J*, the posterior progenitor populations, and *P*, the posterior tubule population. We removed *f*_*JP*_, representing asymmetric fate processes from posterior progenitor to posterior tubule. We also constructed Model 3″, in which the least common (as determined by AUC measurements) fate process in each differentiated population was removed. Therefore, the fate processes presented in Model 3″ were that of *lf*_*JP*_, representing division-independent fate processes of posterior progenitors to posterior tubule cells; *f*_*GA*_, denoting asymmetric fate processes of the anterior progenitors to anterior tubule cells; *f*_*GM*_, representing asymmetric fate processes of the anterior progenitors to MCCs; and *f*_*GP*_, indicating asymmetric fate processes of the anterior progenitors to posterior tubule cells.

### Objective function

To compare outcomes of our simulations to those of known biological data, we constructed an objective function using the mean-squared error of differentiated cell populations across time. For anterior tubule populations, we considered both our own and literature-based cell counts at 20, 48, and 96 hpf and performed a generalized logistic equation fit (Equation 8). The same methodology was applied to the posterior tubule population (Equation 9). For the multiciliated cell population, we gathered data at 20, 28, 48, and 96 hpf and fit values to a logistic equation, similar to those in other populations (Equation 10). In all three equations derived from our regression models, we refer to each as r_X_(*t*), where *‘X’* is a given cell population (*A*, *P*, and *M* for anterior tubule, posterior tubule, and multiciliated cells, respectively).

$$r_{A}\left(t\right)=255+\frac{-255}{{1+\left(\frac{t}{13}\right)}^{3.2}},$$ (8)

$$r_{P}\left(t\right)=411+\frac{-411}{{1+\left(\frac{t}{24}\right)}^{2.9}},$$ (9)

$$r_{M}\left(t\right)=70+\frac{-70}{{1+\left(\frac{t}{15}\right)}^{5.0}}.$$ (10)

Therefore, we arrived at an objective function in which all three equations derived from our regressions were used to compare simulation outcomes with biological data via the mean-squared error (Equation 11).

$$\frac{1}{3n}\sum_{i=1}^{n}\left[{\left(r_{A}\left(t_{i}\right)-{\hat{A}}_{i}\right)}^{2}+{\left(r_{P}\left(t_{i}\right)-{\hat{P}}_{i}\right)}^{2}+{\left(r_{M}\left(t_{i}\right)-{\hat{M}}_{i}\right)}^{2}\right].\;$$ (11)

### Bound algorithmic-based optimization

Our algorithmic-based optimization relied primarily on the usage of a bounded Broyden–Fletcher–Goldfarb–Shanno (BFGS) algorithm, a gradient-based, quasi Newton method (Fletcher, 1987). Other algorithmic-based support was derived from simulated annealing, allowing for broader solution space and avoiding local minima, with a high computational cost (Supplementary Figure S1) (Xiang et al., 2013; Kirkpatrick et al., 1983). For our objective function, we relied upon the mean-squared error of the three statistical models that were synthesized from our own work, along with literature-based values of the anterior tubule, posterior tubule, and multiciliated cell populations from 10–96 h post-fertilization (Equation 10).

For bounding within our parameter optimization, we relied upon both our own data derived from FISH/IF and literature-based values. For proliferation and death of tubule populations, we set our initial parameter estimates to the mean value from our data. For both upper and lower bounds, we extended either 1) two standard deviations from the initial estimates (with the standard deviation derived from our data) or 2) values sourced from literature relevant to the biological process. For proliferation, we used published work to gain an understanding of the cell-cycle duration (which is indicative of the maximum rate of proliferation within a given population) in differentiated cells (Table 2) (Leung et al., 2011).

**TABLE 2:** Zebrafish mature cell population proliferation data.

Mature population	Time in development	Cell-cycle time	G_2_/M length	Source
Retina	28 hpf	6–8 h	65 min	Leung et al. (2011)
Hindbrain	28 hpf	8–10 h	53 min	Leung et al. (2011)

For progenitor populations, we relied more strongly on literature-based values for parameter bounds, referring to cellular populations across tissues and time. As in zebrafish neuronal populations, cell cycles progressively lengthen throughout gastrulation, with cell-cycle lengths reaching up to 4 h (Table 3) (Kimmel et al., 1994). In early (18–26 hpf) zebrafish ocular development, “fast-cycling,” progenitors are generated that undergo divisions every 2–4 h (Table 3) (Tang et al., 2017). However, in later stages of eye development (26–48 hpf), progenitors shift to a “slow-cycling” state, with cell divisions occurring every 12–26 h (Table 3) (Tang et al., 2017). During zebrafish cardiogenesis, progenitors undergo fewer divisions by 48 hpf (Qu et al., 2008; de Pater et al., 2009; Gupta and Poss, 2012; Choi et al., 2013). In zebrafish liver, gut, and other endodermal progenitor populations, cell-cycle lengths last nearly 14 h during early segmentation (12 hpf) (Table 3) (Yang et al., 2021). In other mathematical models of dynamic cell populations, estimates of cell proliferation parameters indicate estimated cell-cycle periods of less than 30 min (Table 3) (Germano and Osborne, 2021).

**TABLE 3:** Progenitor cell-cycle times from the literature.

Progenitor population	Time in development	Cell-cycle time	Source
Neuronal	8 hpf	2–4 h	Kimmel et al. (1995)
Retinal (fast cycling)	12–26 hpf	2–6 h	Tang et al. (2017)
Retinal (slow cycling)	26–48 hpf	16–24 h	Tang et al. (2017)
Liver/gut	12 hpf	13–14 h	Yang et al. (2021)
Modeled progenitors	N/A	0.33–10 h	Germano and Osborne (2021)

Based on this information, we estimated that the maximum turnover rate would be 25% (cell-cycle length 4 h) at any given time (*t*) within our progenitor submodels. This 25% includes symmetric divisions to replenish the progenitor population, asymmetric divisions that maintain progenitor populations while contributing to more mature populations, and finally any division-independent fate processes that occur when a progenitor undergoes a fate change from a multipotent state to a mature state without dividing, which we treated as the sole mechanism by which fate processes occur.

### Monte Carlo-based optimization

In addition to algorithmic-based optimization approaches, we chose to perform a Monte Carlo (MC)-based optimization routine. This method allows us to sample parameter sets that could potentially reside outside any potential minima found within the topology of the objective function. To perform our MC optimization scheme, we synthesized 100,000 parameter sets. For each of these parameter sets, individual parameter values were derived from a uniform distribution, with all parameters constrained by the same biology-based bounds found within our algorithmic-based optimization routine. Then, utilizing the same grid-based approach used in our algorithmic-based approach, we ran each of the 100,000 parameter sets at each of the 10,000 cells found within our grid and recorded the top parameter set for each cell based on the same objective function used in our algorithmic-based approach (Equation 10).

### One-at-a-time (OAT) sensitivity analysis

We selected the single parameter set associated with the lowest MSE/AIC derived from our grid-based (constrained BFGS) optimization scheme. For each parameter analysis, the chosen parameter set was used, and the *ith* parameter was adjusted by ± 5, 10, and 15%. We then recalculated the MSE for this corresponding parameter perturbation, and the process was repeated for *i* parameters (35 in the case of Model 3).

### Sensitivity analysis for initial parameter estimates

Initially, the matrix *A* is constructed, with each row representing the optimized parameter set derived from the constrained BFGS optimization. The primary diagonal of *A* is then scaled by a constant factor *c* (±5%), producing a new matrix ***A'***. Each row of *A*′ is re-optimized using the same constrained BFGS algorithm, resulting in matrix *B*, in which each row contains a re-optimized parameter set based on the scaled values. For each element (*A*_*ij*_) of *A*′, the percent change in the corresponding optimized parameter *B*_*ij*_ from *B* is calculated. This produces matrix *C*, which contains the percent changes in the optimized parameters. Each element of each *ith* row of the matrix *C* now represents the percent change in response to the perturbation of the initial (pre-optimized) parameter value within the *ith* element of the said row. From this row, we assess the influence of the *ith* parameter on the optimization of each non-*ith* parameter, which we term the initial parameter influence (indices) (Supplementary Figure S2A). At the columnar level, each element, where *i ≠ j*, represents the percent change of each *jth* parameter in response to the perturbation of each *ith* parameter, which we term the initial parameter susceptibility (indices) (Supplementary Figure S2B).

### Parameter correlation analysis

To assess potential correlations between model parameters, we computed the Pearson correlation coefficients among the optimized parameter values within the top 100 parameter sets derived from our BFGS-based optimization of Model 3. For visualization, we constructed a correlation heatmap, where a threshold of |r| > 0.4 indicates strongly correlated parameter pairs. Additionally, we examined the impact of correlated parameters on model performance by performing sensitivity analyses and evaluating changes in the objective function when select parameters were perturbed.

### Nested model analysis

In addition to calculating AIC statistics to determine optimal models, we computed the likelihood ratio statistic, which follows a chi-squared (χ^2^) distribution. This property allows us to determine an associated p-value, providing a formal hypothesis-testing framework for assessing whether the inclusion of additional fate-based parameters significantly improved model fit.

### SANN optimization method(s)

For our simulated annealing (SANN)-based optimization scheme, we used a similar grid-based approach to that found in our BFGS-based optimization scheme. We created a 25 × 25 grid (100 × 100 grid in BFGS) of initial progenitor population sizes. We then optimized each of these given combinations (625 in total). We utilized the smaller grid because of the much higher computational cost of SANN-based optimization (Supplementary Figure S1; Supplementary Table S2). All initial parameter values of biological function (proliferation, death, etc.) were kept constant over the two algorithmic-based optimization schemes.

## Results

### Biological parameterizations

**FIGURE 2:**
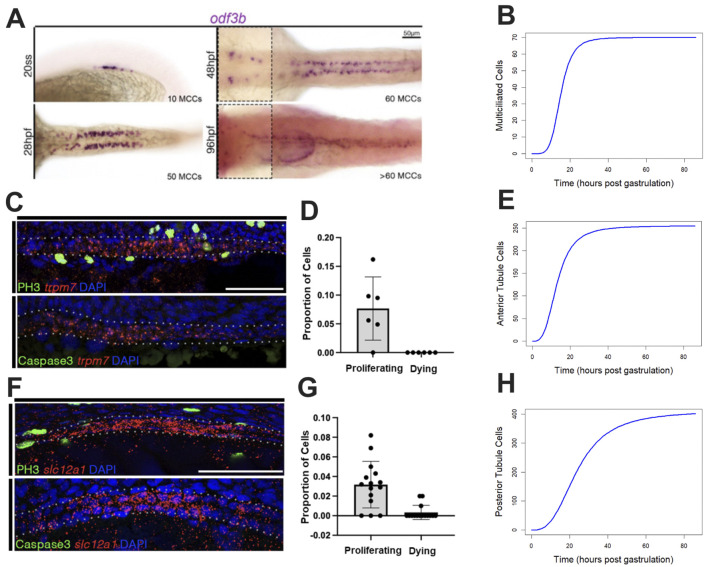
Characterizing the biological properties of populations of interest. **(A)** The MCC populations over time shown via WISH. **(B)** Curve fit of the MCC population over time. **(C,D)** FISH/IF of the PST region (marked by *trpm7*) during early nephrogenesis for determining the proportion of cells undergoing proliferation and death, respectively. **(E)** Curve fit of the proximal tubule population. **(F,G)** FISH/IF of the DE region (marked by *slc12a1*) during early nephrogenesis for determining the proportion of cells undergoing proliferation and death, respectively (DL images found in Supplementary Materials 2). **(H)** Curve fit of the distal tubule population.

To understand cell population dynamics during development, we initially sought to quantify the number of cells within each population throughout development and to determine the frequency of their proliferation and death rates during early developmental stages. To do this, we utilized both our own histological data and data derived from previous publications (Vasilyev, 2009). For the MCC population, we performed WISH using the canonical marker *odf3b*. Cell count was measured at various time points during development. These cell counts were plotted, and a curve was fit to the time-course data (Figures 2A,B; Supplementary Table S3).

For the mature anterior tubule population, we performed dual FISH/IF to 1) determine the number of cells present in the region of interest and 2) gain a better understanding of the intrapopulation dynamics such as proliferation. We used FISH to count the number of *trpm7+* cells present in the early nephron. Using the canonical PST marker *trpm7*, we created an index of cell density that was used to generalize the proximal region of the developing nephron, allowing us to better understand the whole proximal tubule. We performed IF using the canonical proliferation marker PH3 (phospho-histone H3) to determine the number of cells in the G2 or M phase of the cell cycle at 20 hpf (Figures 2C,E; Supplementary Table S3). We performed IF using the canonical apoptotic marker Caspase3 to determine the number of cells undergoing apoptosis at 20 hpf (Figures 2C–E; Supplementary Table S3).

For the mature posterior tubule populations, similar to the anterior tubule population, we performed dual FISH/IF to determine the number of cells present within the population at 20 hpf, along with the number of cells undergoing proliferation and death at the same time point. We performed our proliferation and death assays on both segments of the distal region of the nephron (DE and DL; Figure 1A; Supplementary Table S3). For the DE segment, we utilized the marker *slc12a1* in conjunction with the proliferation and death markers (Figures 2F–H; Supplementary Table S3). For the DL studies, we utilized the marker *slc12a3* (Supplementary Figure S2B; Supplementary Table S3).

### Investigation of differential fate processes

**FIGURE 3:**
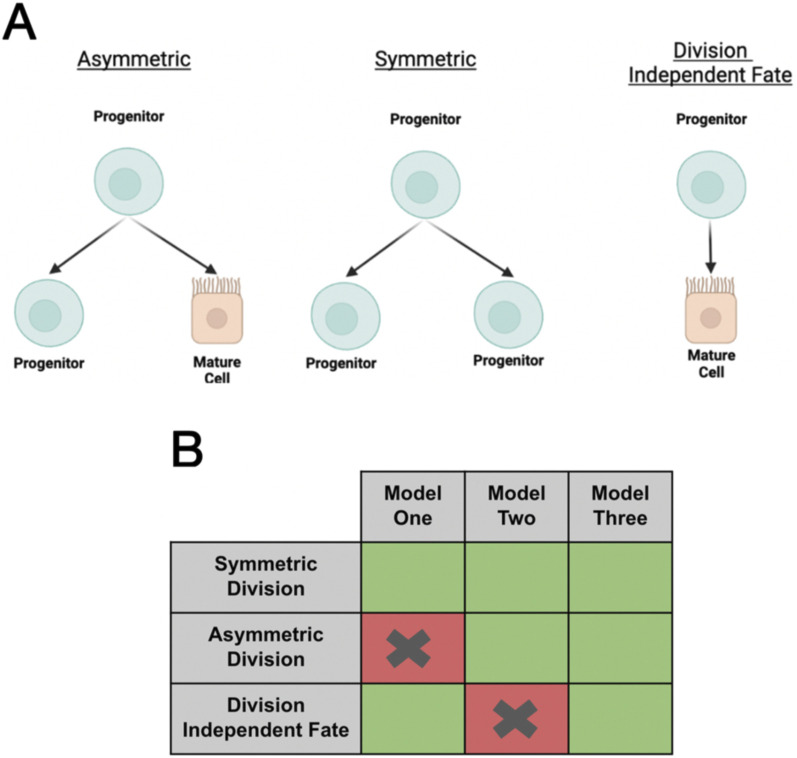
Submodels involved in progenitor populations. **(A)** Illustration of stem cell dynamics via asymmetric, symmetric, and division-independent fate processes. **(B)** Graphic demonstrating each of the progenitor models discussed in this study. **(C)** Outcome of the initial optimization scheme with models under the assumption of fixed parameters/cellular characteristics.

In the zebrafish model, the fate of the cells that form the functional units of the kidney is not well understood in a mechanistic sense. However, in the rat model, after ischemic injury, damaged cells dedifferentiate to form progenitor-like cells, which then undergo asymmetric divisions to restore the tubule epithelium (Maeshima et al., 2003). This provides evidence that asymmetric divisions occur in an *in vivo* model of renal repair. A more contemporary literature derived from human fetal samples suggests that nephron progenitor cells may have a more nuanced trajectories, including fates to proliferative cell niches being independent of a semi-differentiated proximal/distal precursor(s) (Lindström et al., 2018). In a mouse (*in vitro*) model, NPCs have shown the ability to 1) successfully undergo self-renewal and 2) retain physical homology with other NPCs (Huang et al., 2025). However, in the zebrafish pronephric system, such resolution and information regarding progenitors remain unknown. Therefore, to determine the potential relationship(s) between the mature populations previously described and the progenitor populations, we decided to test three different hypotheses for determining the mechanism by which mature cells arise in the zebrafish pronephros. In our first full model, we would test the hypothesis that progenitors undergo fate decisions via division-independent means, which are independent of proliferation, a mechanism of differentiation that has been shown across animal and tissue models (Figures 3A,B) (Snippert et al., 2010; Reilein et al., 2018; Ismaeel et al., 2024; Bessonov et al., 2019). Our second full model would consist of the more classical understanding of stem or stem-like cells, in which a resident progenitor population undergoes asymmetric differentiation, where cell fate and proliferation are a completely dependent process (Figures 3A,B). Our third model would have both asymmetric and division-independent fate processes, allowing us to determine whether both differentiation types can contribute to a viable model for nephron development in the zebrafish pronephros. Through our optimization strategy, in each of these models, we believe that we can infer potential properties of progenitor populations, particularly the rates at which proliferation occurs across pronephros development.

To answer these questions, we utilized a grid-based optimization algorithm-backed strategy. To determine rates at which progenitors and mature cells proliferate, die, and undergo fate processes over time, we utilized algorithmic-based optimization under a constrained BFGS paradigm, where constraints were placed based on our biological understanding of each of the cell populations through experimental observations or literature-based values.

**FIGURE 4:**
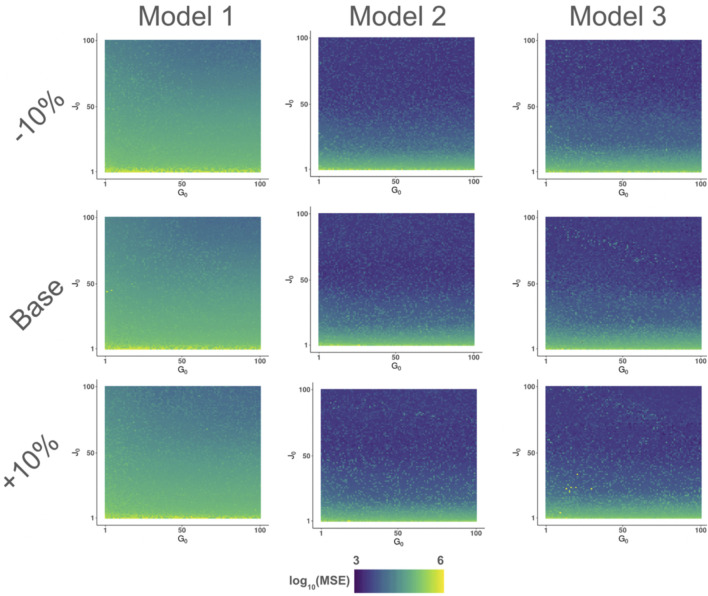
Use of a grid-based algorithmic search for optimal parameter sets/initial progenitor counts. Models 1–3 referenced with respect to Figure 3B. Base parameter estimates represent initial estimates supplied to the optimization scheme.

To determine the most likely number of progenitors in each population at time zero (10 hpf), we utilized a grid-based search, where we optimized at each combination of progenitors from 1 to 100 cells of each progenitor population, resulting in a 10,000-cell grid. To take precautions with respect to initial values within our optimization routine(s), we adjusted all of our initial parameter estimates by a factor of ±10% to gain a better confidence level in the results of our parameter optimizations (Kelley, 1999). Using both of these strategies, we optimized all three models at three different (initial) parameter estimates, resulting in a 3 × 3 arrangement of 100 × 100 grids for the initial progenitor population(s) (Figure 4).

Using our grid-based optimization procedure, we were then able to procure the optimum parameter sets for each of the three proposed models and compute summary statistics for each of these optimized sets (Table 4). Using this information, we applied Akaike information criterion (AIC) and determined that Model 3, which contains fate processes that are both division-dependent and -independent, was the most likely to occur in early renal development (Akaike, 1974). In addition to identifying Model 3 as the most likely model, we also obtained the parameter estimates for each parameter within our model (Table 5).

**TABLE 4:** Relevant diagnostic values for models 1–3.

Model	Minimum MSE (log_10_)	MSE [95% CI] (log_10_)	Minimum AIC	LRT statistics	LRT associated (P-value)
1	3.88	[4.74, 4.76]	3083	571	1.83 ✕ 10^–123^
2	3.11	[3.72, 3.75]	2625	113	1.27 ✕ 10^–24^
3	2.92	[3.76, 3.79]	2530		

**TABLE 5:** Optimized parameters (from Model 3) and their 95% confidence interval (confidence intervals based on top 100 parameters).

Parameter	Optimal [95% CI]	Parameter	Optimal [95% CI]
lA_max	0.01 [0.02, 0.03]	lfGM_width	49.78 [49.77, 49.89]
lA_time	26.05 [18.08, 19.68]	d_E	0.00 [0.00, 0.00]
lA_width	54.68 [66.42, 70.95]	d_P	0.00 [0.00, 0.00]
lP_max	0.01 [0.02, 0.02]	fGP_max	0.12 [0.10, 0.12]
lP_time	39.11 [30.81, 33.01]	fGP_time	16.90 [14.00, 15.36]
lP_width	105.34 [112.76, 115.45]	fGP_width	61.65 [65.07, 67.11]
fGA_max	0.18 [0.19, 0.20]	lfGP_max	0.01 [0.02, 0.02]
fGA_time	12.19 [8.70, 9.21]	lfGP_time	15.86 [17.81, 18.53]
fGA_width	52.57 [63.98, 68.04]	lfGP_width	50.80 [51.12, 51.72]
lfGA_max	0.06 [0.03, 0.04]	fJP_max	0.10 [0.06, 0.07]
lfGA_time	13.62 [14.78, 16.76]	fJP_time	6.73 [10.47, 11.98]
lfGA_width	49.95 [51.00, 51.91]	fJP_width	32.47 [38.78, 41.47]
fGM_max	0.08 [0.07, 0.08]	lfJP_max	0.14 [0.11, 0.12]
fGM_time	16.95 [15.99, 16.81]	lfJP_time	23.65 [18.87, 20.18]
fGM_width	50.49 [51.34, 51.87]	lfJP_width	53.17 [59.54, 62.37]
lfGM_max	0.01 [0.03, 0.04]	lG_max	0.19 [0.22, 0.22]
lfGM_time	16.84 [20.07, 20.86]	lG_1	3.02 [3.02,3.03]
		lG_2	4.97 [4.97, 4.98]

As optimized parameters are indeed sensitive to initial parameter estimates, we also aimed to characterize how alterations in our initial parameter guesses within our model would result in changes in optimized values. To assess this phenomenon in our model, we performed two individual sensitivity analyses of the initial guesses, allowing us to determine the initial parameter influence and susceptibility (Supplementary Figures S2A,B). From this initial parameter sensitivity analysis, we found that parameters associated with death underwent the greatest alteration in relation to the parameter guesses supplied to our constrained BFGS. As for parameters that have the greatest influence or the ability to alter the optimization of other parameters, we found that parameters associated with duration over which fate processes can occur had the highest rating. This work also highlighted the potential for equifinality across our parameter sets within Model 3. Therefore, we implemented the simulated annealing (SANN) algorithm, an optimization method with capabilities to escape local minima within the topology of objective functions (Kirkpatrick et al., 1983). We found no discernable difference in optimizations utilizing SANN and BFGS (Supplementary Figure S3).

In addition to the algorithmic-based optimization procedure, we also performed a MC simulation-based optimization procedure to sample a wider range of parameters, as they were not subject to potential local minima found in our objective function. Similar results were obtained while utilizing said MC simulation, with Model 3 yielding the highest performing parameter sets (Supplementary Table S4).

### Interrogation of optimized parameter sets

**FIGURE 5:**
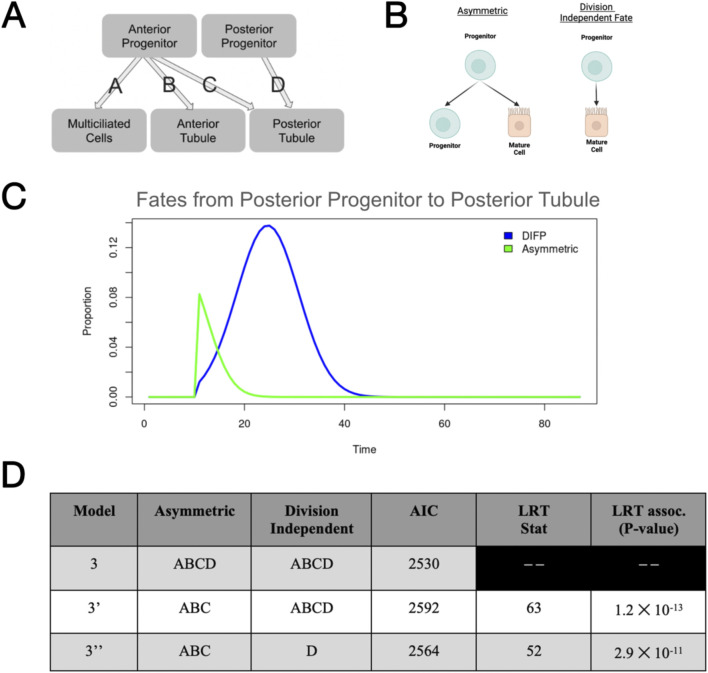
Investigation of the best fitting model(s), with emphasis on fate processes. **(A)** Representation of our model with labeling of fate processes that are investigated in Supplementary Figure S5. **(B)** Representation of the two forms of fate processes found in the Model 3 paradigm.**(C)** Follow-up of both division-independent and asymmetric fate processes in the relationship between posterior progenitors and the posterior tubule population (taken from mean parameter values within the top 100 models). **(D)** Summary of information on reduced model comparisons including LRT statistics and the associated P-values.

As our optimization scheme projected that both forms of fate processes occur in our models, we were then interested in examining the likely temporal and quantitative distributions of each of these fate processes throughout development. We used the top 100 parameter sets from our Model 3 parameter estimates generated by our optimization scheme, representing the top 1% of all parameter sets. We then calculated the amount of each type of fate processes originating from each relationship between progenitor and progeny and then quantified the resulting area under the curve (AUC) (Figures 5A,B).

Based on this, we concluded that in all relationships derived from the anterior progenitor population (*G*), asymmetric or division-dependent fate processes occurred at a higher rate throughout development (Supplementary Figures S4A–C). Interestingly, in our analysis of parameter sets, we found that in the relationship between posterior progenitors and posterior tubule (*J* and *P*, respectively), division-independent fate processes occurred at higher rates in early development (Supplementary Figure S4D). Upon analysis of the temporal distribution of these two forms of fate processes, we found that division-dependent fate processes were likely to occur from the genesis of the posterior tubule populations, whereas division-independent fates were likely to occur at later developmental stages, but with much greater magnitude and for a longer duration of development (in the top 100 performing parameter sets) (Figure 5C). This idea of temporal differences in fate processes across related populations is not novel in the mammalian model, as previous work has shown evidence for dissimilar induction of fate processes across renal populations (Lindström et al., 2018).

To determine whether differential forms of fate processes are required during renal development, we reevaluated the composition of Model 3, which includes both division-dependent and division-independent fate processes among all relationships between progenitor and progeny. As our previous analysis revealed that division-independent fate processes are the primary source of differentiation within the posterior progenitor (J), we synthesized two additional submodels from model 3, 3′ and 3′′. We then (using the initial parameter guesses given in our initial optimization scheme) optimized each of these new models using the same optimization scheme previously described in our initial optimization experiments. We then compared the best parameter set for each paradigm. We utilized both AIC and the likelihood-ratio test (LRT) to compare each of these three models/parameter sets. From our analysis, we determined via both AIC and LRT that differential forms of fate processes in both anterior and posterior progenitors and all ensuing relationships between anterior, posterior, and multiciliated cells are indeed a possibility (Figure 5D).

As it seemed that differential forms of fate processes were integral to model success, we aimed to survey all parameters within Model 3 and determine whether other biological phenomena were equally (or more) vital for model success. To gain this insight into the importance of individual parameter values, we performed OAT sensitivity analysis. Our OAT sensitivity analysis revealed that parameters associated with proliferation of mature populations, not fate processes, substantially alter outcomes of the optimized model (Supplementary Figure S5). We also observed that proliferative maximum of the progenitor populations (lG_max_) was somewhat sensitive as a reduction in this parameter resulted in poorer model fit, aligning with previous *in silico* and *in vivo* studies (Cebrián et al., 2004; Zubkov et al., 2015).

**FIGURE 6:**
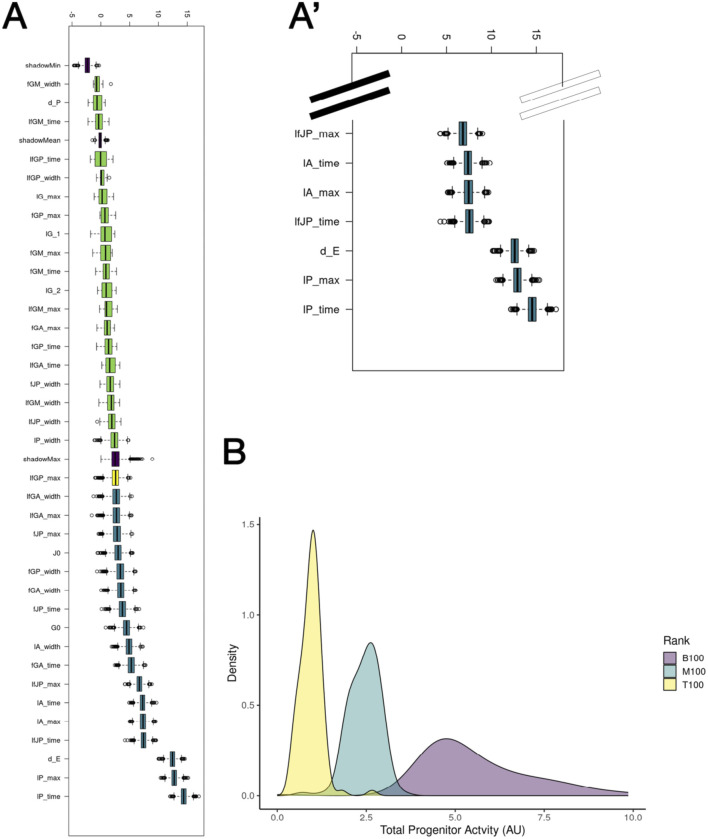
Investigation of individual parameters in Model 3. **(A)** Boruta analysis of feature selection highlights the proliferation of the posterior tubule population. **(A′)** Inset of A showing top seven features selected via Boruta. **(B)** Area under the curve for highest-, medium-, and lowest-performing 100 parameter sets with respect to progenitor activity.

To further investigate model parameters and their associations with model success, we performed Boruta analysis to determine which model parameters are most closely associated with model success in the Model 3 paradigm (Figures 6A,A’). Similar to our OAT sensitivity analysis, the parameters most closely associated with model success were those related to proliferation (lP_time_ and lP_max_, representing the time during which proliferation of the posterior tubule peaks and the maximum proliferative rate of the posterior tubule population, respectively). However, interestingly, the parameters of division-independent fate processes, along with, perhaps most surprisingly, death rates of anterior tubule (*A*), MCC (*M*), and progenitor populations (*G/J*), were found to be among the most indicative of model performance. These results indicate that proliferation of mature populations, not progenitor populations, may play a key role in proper renal development.

To follow up on this, we utilized the 10,000 parameter sets obtained from our optimization scheme to determine the necessity of active (with respect to division and differentiating) progenitor populations in early renal development. We selected the 100 highest-, medium-, and lowest-performing parameter sets from our optimization scheme and analyzed their resulting distributions with respect to the area under the curve of the two progenitor populations over time (Figure 6B; Equation 12).

$$Progenitor\;Activity=\int_{t_{0}}^{t_{86}}\left(G+J\right)\;dt.$$ (12)

We found that parameter sets in which progenitors were less likely to undergo fate processes and proliferation were more closely associated with model success, indicating that mature, not progenitor, populations are more likely to be active during early renal ontogeny. This trend from our parameter sets derived from the top 100/10,000 parameter sets supports the hypothesis that differentiated cell populations may be more mitotically active than their precursors, with multiple studies suggesting that after injury mature tubule populations undergo proliferative responses to aid in the processes of regeneration (Vogetseder et al., 2008; Kusaba et al., 2014; Brilli Skvarca et al., 2019). To examine the relationship between progenitor activity and parameter set outcomes, we next analyzed the distribution of the initial number of progenitors (G_0_ + J_0_) across the 100 highest-, medium-, and lowest-performing parameter sets and found that the best parameter sets were strongly associated with higher initial progenitor counts (Supplementary Figure S6).

To further explore the potential for equifinality, we also addressed correlations between model parameters by conducting a parameter correlation analysis of the top 100 parameter sets derived from BFGS. Pearson correlation coefficients applied among all optimized parameter values identified interdependencies that might indicate redundancy or non-identifiability. Strongly correlated parameter pairs (|r| > 0.4) were visualized using a heatmap (Supplementary Figure S7). In our analysis of these correlated parameter pairs, many correlations were considered (biologically) irrelevant. For example, our generalized death parameter and the maximum proportion of the anterior progenitor population that can undergo a symmetric fate process, where one cell becomes a posterior tubule, were found to be (negatively) correlated with one another. However, there were instances where two (correlated) parameters were found to suggest some degree of equifinality within our model. For example, the parameters associated with the width of fate transitions (division-independent and division-dependent) from posterior progenitors to the posterior tubule were found to be highly correlated. This suggests that a level of equifinality exists with respect to our model.

## Discussion

Our work presented here provides a comprehensive and computationally rigorous framework for understanding zebrafish nephrogenesis, demonstrating the promise and power of mathematical modeling in development. By integrating empirical data with mathematical modeling, we have advanced our understanding of how progenitor and mature cell populations interact and contribute to the development of the nephron within the zebrafish model. In particular, and perhaps most importantly, we have demonstrated the hypothetical necessity of both division-dependent and division-independent fate processes in early renal development, offering potential insights into the plasticity of cell fate decisions during this delicate time period in development, a feature known to exist in the genesis of other organs (Snippert et al., 2010; Reilein et al., 2018; Ismaeel et al., 2024).

Our model provides new perspectives on qualities and relationships between progenitor and progeny cell roles in nephrogenesis. Our hypothesis that mature populations play a more significant role in early nephron development than previously thought adds a new layer of complexity to our understanding of renal development. This observation suggests that rather than a strict, linear progression from a progenitor to differentiated cell, there may be more fluid interactions between these populations, as has been shown in regeneration (Okamura et al., 2021). This idea of maturated/differentiated cells being responsible for growth is not new as in regeneration, differentiated populations act as drivers of neo-nephrogenesis (Berger and Moeller, 2014; Brilli Skvarca et al., 2019; Liu et al., 2023). During development, progenitor cells are described as the “slower-cycling population,” with ureteric tip cells having markedly faster cell-cycle times than their cap mesenchyme counterparts (Short et al., 2014). Our work aligns with this hypothesis of active differentiated cells being responsible for growth during zebrafish nephrogenesis. Further support for this perspective encourages the development of new experimental hypotheses, including exploring the transcriptional profiles of both populations among multiple possible fate mapping experiments in both zebrafish and other models.

Moreover, our use of MC simulations, combined with our constrained optimization procedure in both BFGS and SANN paradigms, strengthens the reliability of our conclusions made from the modeling-based experiments. These approaches, although computationally demanding, are robust and invaluable for providing a comprehensive exploration of the parameter space, allowing us to make inference(s) upon optimization outcomes. Furthermore, our grid-based optimization routine via both algorithmic and Monte Carlo methods, where we searched for optimal progenitor population values, allows for a more thorough exploration of possible starting conditions.

The ability to predict and possibly explore behavior within the progenitor populations responsible for the development of the first nephron(s) *in silico* offers clear advantages over purely experimental approaches, which are limited by technical and biological factors. By coupling experimental data with computational models, we have established a potentially iterative process, which allows us to ask and refine biological questions via computational means and then provides experimental results.

We believe that the implications of the work extend into the broader developmental biology and nephrology communities. One key insight from our studies is that we present and provide sufficient evidence for the possibility of division-independent fate processes being a necessity of proper pronephros development. In the context of nephrogenesis during development and neo-nephrogenesis during regeneration, this opens exciting avenues for further experimentation of the relationship between transcriptional mechanisms, proliferation, and fate processes. We believe that this general framework can also be applied to developmental processes with the ability to explore additional avenues such as gene regulation and mechanical forces. Our goal was to build a hypothetical characterization of the role and properties of progenitors during early renal development in the zebrafish model. However, additionally, our model brings forth interesting perspectives that could be explored from an evolutionary perspective. Plasticity between progenitor and fully differentiated cells among renal populations across other vertebrate species also presents as an exciting avenue for future exploration, an idea of current interest (Piedrafita et al., 2020).

## Limitations of the model and avenues for future exploration

Despite the strengths of this study, several limitations must be acknowledged. First and foremost, the assumption of constant death rates for the different cell populations is a simplification that may not capture possible dynamic regulation of apoptosis during development. Incorporating more dynamic death rates could provide a more accurate representation of cellular turnover; however, in our OAT sensitivity analysis, we reveal that cellular death processes contribute minimally to model outcomes (Supplementary Figure S5). Future iterations of the model could explore more complex, time-varying death processes that better reflect the biological realities of cellular dynamics, with the addition of genetic regulatory networks that govern items such as death and proliferation, requiring further experimental and computational experimentation.

Second, we acknowledge the potential for equifinality within our model, particularly in relation to our objective function. This equifinality is most noticeably displayed within our BFGS-based optimization grids, where the result (MSE) shows no noticeable pattern/gradient on the graph (Figure 4). Although we have taken steps to mitigate this by altering initial parameter values within our grid search, utilizing pre-optimization sensitivity analysis, both algorithmic and nonalgorithmic optimization schemes (via MC simulations), some degree of equifinality may persist, reflecting both the complexity and potential points for flexibility within the zebrafish pronephros. Despite the evidence for equifinality, our conclusions regarding 1) the potential for division-independent fate processes and 2) the relative inactivity of progenitor populations after early development are supported by empirical evidence from our survey of parameter sets associated with the 100 highest-, medium-, and lowest-performing parameter sets derived from algorithmic-based optimization, with follow-up insights from progenitor activity AUC analysis. Future directions for better understanding the level of equifinality or methods to gain further insights into mechanistic qualities of progenitors such as proliferative capacity can be found via Bayesian approaches (Coelho et al., 2011; Linden-Santangeli et al., 2025a; Linden-Santangeli et al., 2025b). Other methods, such as spatially explicit models, have provided insights into complex morphogenetic processes, including in zebrafish (Qiu et al., 2021).

Similarly, although the model includes fate processes as dynamic and integral to cell differentiation, the molecular pathways driving these processes remain not entirely understood in zebrafish nephrogenesis. As previously mentioned, the inclusion of molecular drivers in future models would allow for a more detailed and biologically interpretable framework, potentially enabling the identification of key signaling pathways that regulate fate processes during development (Qiu et al., 2021). This could provide deeper insights into how fate decisions are made at the cellular level and how these decisions can be manipulated for more translational purposes.

Another limitation of the current study is the lack of spatial modeling. The exclusion of spatial factors indicates that we do not account for potential gradients of signaling molecules or the mechanical forces that may influence progenitor and/or differentiated cell behavior within each of the distinct cellular populations. Other studies utilizing mouse-derived data for spatial modeling have been utilized and have provided key insights into morphogenesis in the mammalian model (Lambert et al., 2018; Menshykau et al., 2019; Cook et al., 2021). However, this body of work further supports the need for agent-based modeling within the field of renal cell specification, along with spatial transcriptomic studies to further understand the spatially dependent transcriptional process that govern/regulate cellular-level actions/interactions in zebrafish pronephros development. Along with the limitations of not utilizing spatial models, we also acknowledge that we have not included all populations found within the zebrafish pronephros; the cloaca, interrenal gland, podocytes, and corpuscles of Stannius were all excluded from our models.

Taken together, despite the limitations of *in silico* modeling, the inferences gathered from our simulation studies and follow-up analyses support further rigorous examinations of pronephros development in the zebrafish and other traditional/non-traditional models.

## Data Availability

The original contributions presented in the study are included in the article/Supplementary Material; further inquiries can be directed to the corresponding authors.

## Abbreviations

AUC, area under the curve; BFGS paradigm, Broyden–Fletcher–Goldfarb–Shanno paradigm; CS, corpuscle of Stannius; DE, distal early; DL, distal late; FISH, fluorescent *in situ* hybridization; IF, immunofluorescence; hpf, hours post-fertilization; MC, Monte Carlo; LRT, likelihood-ratio test; MCC, multiciliated cell; NPCs, nephron progenitor cells; OAT sensitivity analysis, one-at-a-time sensitivity analysis; PCT, proximal convoluted tubule; PST, proximal straight tubule; SANN, simulated annealing; SS, *s*omite stage; WISH, whole-mount *in situ* hybridization; WT, wild-type.
