# Supplementary material for: Mathematical modeling reveals cell differentiation processes and progenitor kinetics necessary for proper nephrogenesis
Source: Front Cell Dev Biol. 2025 Dec 11;13:1695380. doi: 10.3389/fcell.2025.1695380 (PMC12738939; doi:10.3389/fcell.2025.1695380)
Supplement: Supplementary file 1 [file DataSheet1.docx]

**Supplemental Figures/ Tables/ Methods**

| **Reagent or Resource** | **Name** | **Source** | **Catalog Number** |
| --- | --- | --- | --- |
| Antibodies | Anti-Ph3 | Millipore | 06–570 |
|  | Anti-Caspase3 | BD Biosciences | 559565 |
|  | Goat anti-Mouse, Alexa Fluor 568 | Invitrogen | A11031 |
|  | Goat anti-Rabbit, Alexa Fluor 594 | Invitrogen | A11037 |
|  | Goat anti-Mouse, Alexa Fluor 488 | Invitrogen | A11029 |
|  | Goat anti-Rabbit, Alexa Fluor 488 | Invitrogen | A11034 |

**Supplementary Table 1.**  Antibodies used in proliferation and death assays.

| **Method** | **Run-Time (CPU clock in Days)** | **Convergence Rate** | **log10(MSE) of top parameter set** |
| --- | --- | --- | --- |
| BFGS | 133 | 99.7% | 2.92 |
| Monte Carlo | 238 | NA | 4.73 |
| SANN | 887* | 100%** | 3.02 |

**Supplementary Table 2.** Optimization Summary Metrics. ***** SANN optimization utilizes a 25×25 optimization scheme, where BFGS and Monte Carlo based schemes were 100×100. ****** SANN termination determined via tolerance-derived stopping with a predetermined tolerance point, as GenSA does not provide a conventional convergence code (Xiang et al., 2013).

| **Population** | **Time**  **(hpf)** | **Number of Cells (Proliferating \| Dying)** | **Citation**  **(If Applicable)** |
| --- | --- | --- | --- |
| Anterior Tubule | 20 | 94 (6 \| 0) | -- |
| Anterior Tubule | 36 | 208 | Vasilyev et al., 2009 |
| Anterior Tubule | 96 | 242 | Vasilyev et al., 2009 |
| Posterior Tubule | 20 | 83 (2 \| 0 ) | -- |
| Posterior Tubule | 36 | 211 | Vasilyev et al., 2009 |
| Posterior Tubule | 96 | 411 | Vasilyev et al., 2009 |
| MCC | 20 | 10 | -- |
| MCC | 24 | 25 | -- |
| MCC | 28 | 50 | -- |
| MCC | 48 | 60 | -- |
| MCC | 96 | 75 | -- |

**Supplementary Table 3**. Characteristics/ cell counts of maturated cell populations (Shown as median).

| **Model** | **Minimum MSE (log_10_)** | **Minimum AIC** |
| --- | --- | --- |
| 1 | 4.88 | 3680 |
| 2 | 4.83 | 3652 |
| 3 | 4.73 | 3633 |

**Supplementary Table 4.** Relevant Diagnostic Statistics from MC based optimization


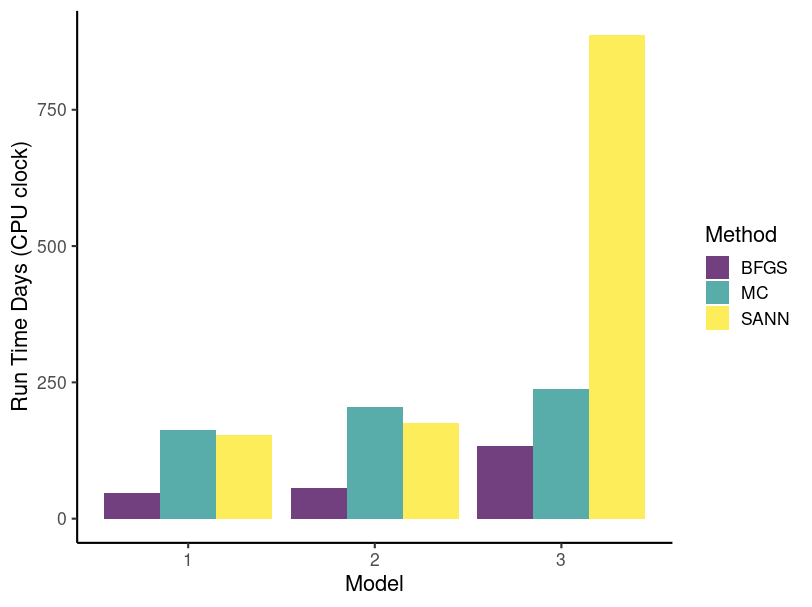


**Supplementary Figure 1.** Comparison of computational cost across algorithms/ Monte Carlo simulations and models in terms of CPU run time days*. *Note that SANN optimization was utilized on a 25x25 optimization scheme (625 runs), while BFGS/ MC was utilized on a 100 x 100 scheme (10,000 runs)

**
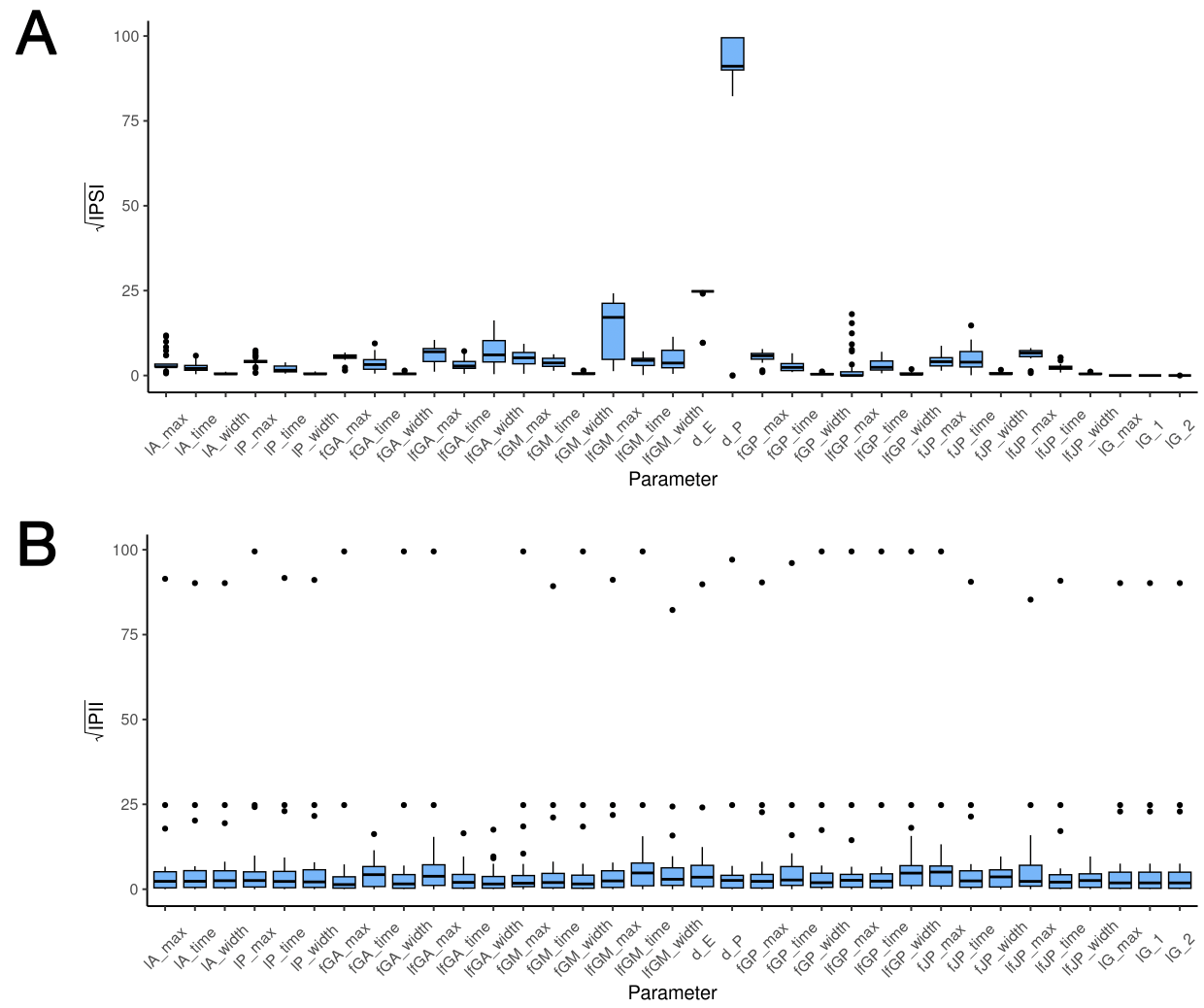
**

**Supplementary Figure 2.** Initial parameter sensitivity analysis. A) Initial parameter susceptibility index plot showing parameters likely to have greatest (post optimization) changes in relation to initial guesses supplied to BFGS algorithm. B) Initial parameter influence index plot showing parameters which change (pre optimization) is to cause fluctuations across other parameters (post optimization)


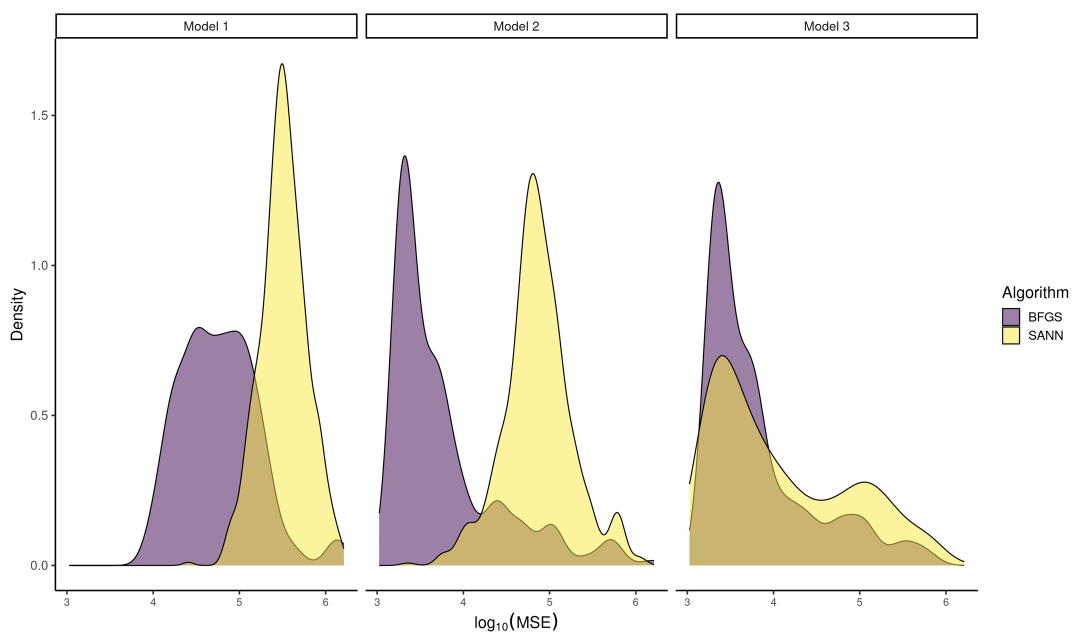


**Supplementary Figure 3. Comparison of SANN and BFGS derived optimizations**. Comparison of log_10_(MSE) across each model by optimization algorithm utilized.


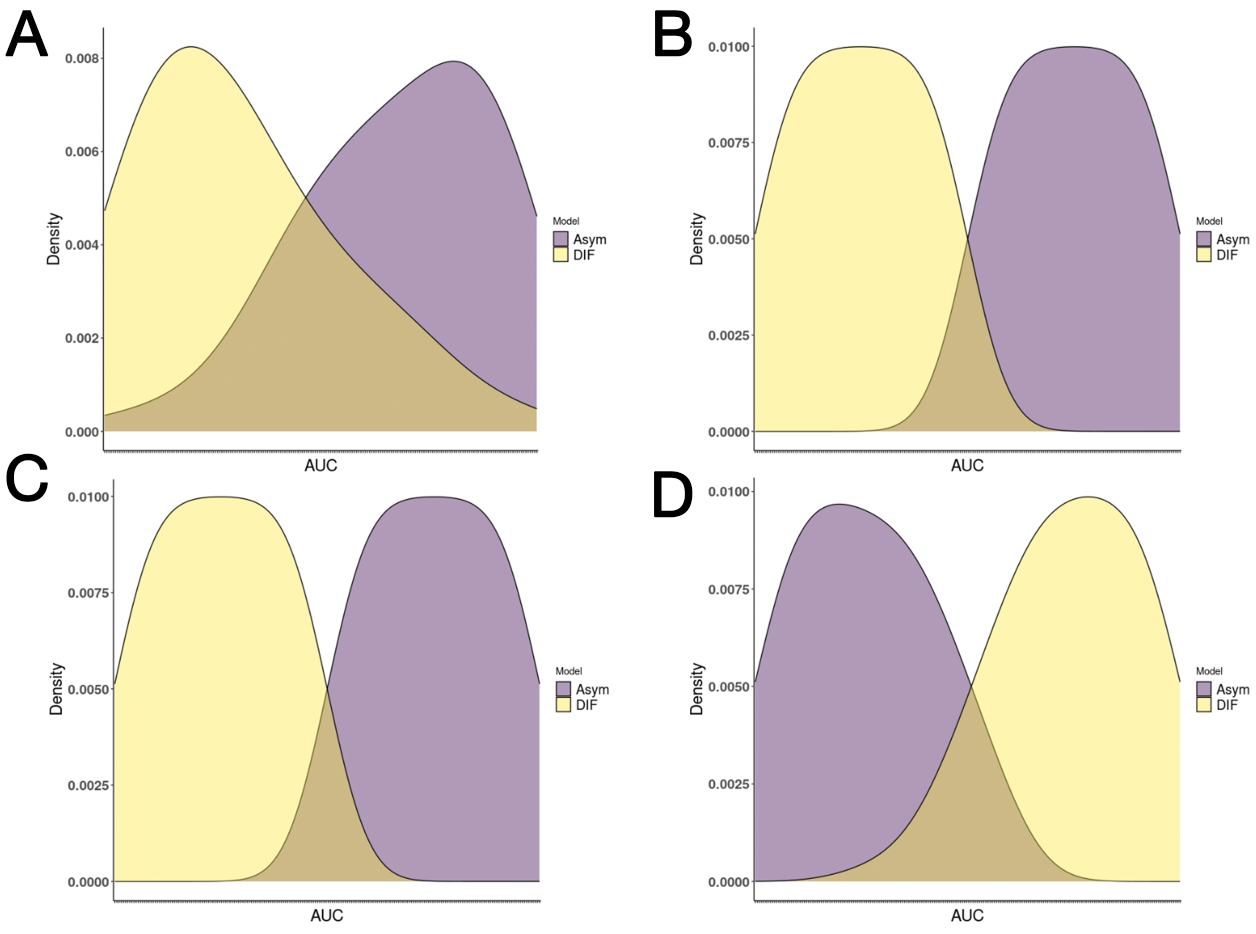


**Supplementary Figure 4. Area Under the Curve Measurements for Fate Processes derived from Top 100 Parameter Sets of Model 3. A) Anterior Progenitor to MCC B) Anterior Progenitor to Proximal Tubule C) Anterior Progenitor to Distal Tubule D) Posterior Progenitor to Distal Tubule**

**
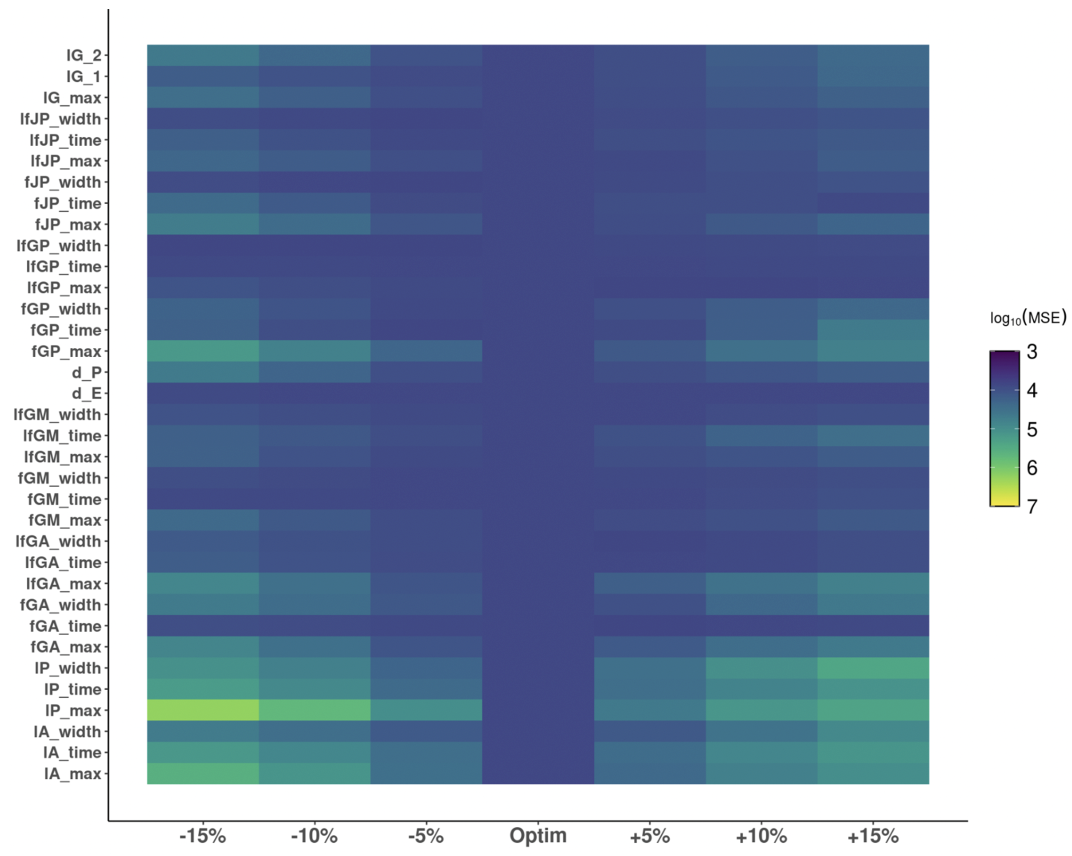
**

**Supplementary Figure 5.** One-at-a-time sensitivity analysis of parameters found in model 3.


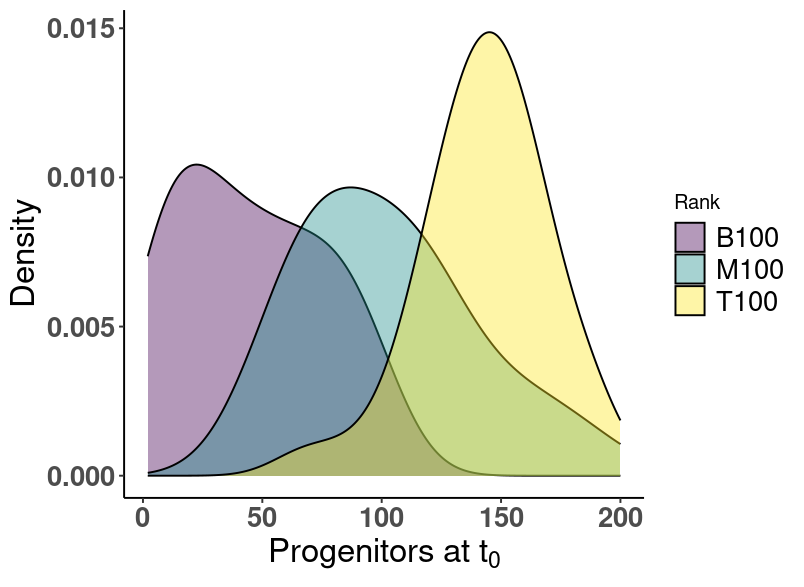


**Supplementary Figure 6.** Distribution of Initial number of progenitors (G_0_ + J_0_) by model rank.

**
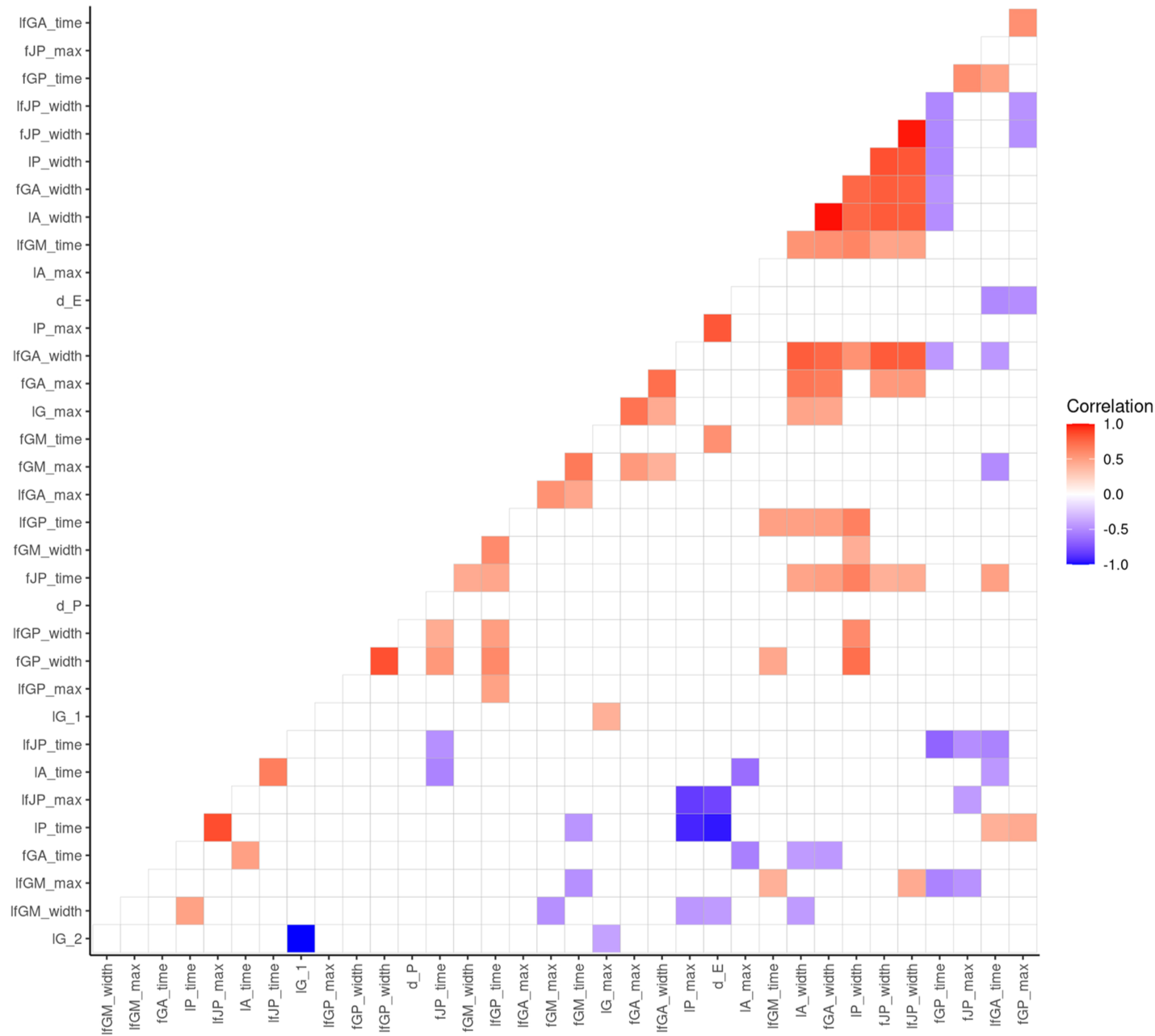
**

**Supplementary Figure 7.** Correlation between parameters derived from top one-hundred parameter sets from BFGS optimization scheme. Cutoff for Pearson correlation set to > ± .4 for heatmap shown.
